# Influence of MTHFR Genetic Background on p16 and MGMT Methylation in Oral Squamous Cell Cancer

**DOI:** 10.3390/ijms18040724

**Published:** 2017-03-29

**Authors:** Nadia Ferlazzo, Monica Currò, Angelo Zinellu, Daniela Caccamo, Gaetano Isola, Valeria Ventura, Ciriaco Carru, Giovanni Matarese, Riccardo Ientile

**Affiliations:** 1 Department of Biomedical and Dental Sciences and Morphofunctional Imaging, University of Messina, 98124 Messina, Italy; nferlazzo@unime.it (N.F.); moncurro@unime.it (M.C.); dcaccamo@unime.it (D.C.); gisola@unime.it (G.I.); valeriaventura82@hotmail.it (V.V.); gmatarese@unime.it (G.M.); 2 Department of Biomedical Sciences, University of Sassari, 07100 Sassari, Italy; azinellu@uniss.it (A.Z.); carru@uniss.it (C.C.); 3 Control Quality Unit, University Hospital of Sassari (AOU), 07100 Sassari, Italy

**Keywords:** oral squamous cell cancer, *MTHFR* polymorphisms, global DNA methylation, *p16* promoter methylation, *MGMT* promoter methylation

## Abstract

Genetic polymorphisms of the methylenetetrahydrofolate reductase (MTHFR) enzyme may influence DNA methylation. Alterations in DNA methylation patterns of genes involved in the regulation of the cell cycle, DNA repair, cell adherence and metastasis process are known to contribute to cancer development. In this study, the influence of the MTHFR C677T and A1298C gene polymorphisms on global DNA methylation and site-specific methylation on *p16* and *O*^6^-methylguanine-DNA methyltransferase (*MGMT*) gene promoters was investigated in patients with oral squamous cell cancer (OSCC). To this aim, methylation studies were carried out by using genomic DNA isolated from saliva samples of 58 OSCC patients and 90 healthy controls. The frequency of the CT/AC and TT/AA genotypes was significantly higher in patients than in controls. Whereas no difference in global DNA methylation levels was observed between patients and controls, a higher frequency of methylation at both *p16* and *MGMT* gene promoters was detected in patients compared with controls. A significant association between *MTHFR* gene polymorphisms and *p16* and *MGMT* gene promoter methylation was found. The frequency of *p16* and *MGMT* methylation was around 60% in patients with either the CT/AC or TT/AA genotype. Our results suggest that hypermethylation of cancer-related genes may be affected by *MTHFR* polymorphisms.

## 1. Introduction

Head and neck cancer (HNC), representing the sixth most common cancer worldwide [1,2], encompasses a heterogeneous group of aggressive epithelial malignancies, more than 90% of which are squamous cell carcinomas (SCC).

Oral SCC (OSCC) is one of the most common types of HNC, with a considerable incidence of new cases every year. OSCC more frequently affects men than women (M:F = 2:1). The probability of developing OSCC increases with the period of exposure to risk factors, represented by a diet low in fresh fruits and vegetables, poor vitamin intake, alcohol consumption, and abuse of tobacco smoking [3,4]. Moreover, infection with high-risk human papillomavirus genotypes has also recently been implicated in the etiopathogenesis of OSCC [5].

In recent years, hereditary factors as well as age-related mutagenic and epigenetic changes have been involved in the development of OSCC [6,7].

Epidemiological studies have shown that deficiency of specific nutrients, such as folate, may increase the risk of OSCC [8,9]. Folate is a key source of the one-carbon group used for DNA methylation, the most important form of epigenetic modification, which consists of the addition of one methyl group on a cytosine that precedes a guanine, so-called CpG dinucleotides, and is critical for normal genome regulation and development [10,11]. Global hypomethylation in genomic DNA as well as hypermethylation in specific gene promoters are common features occurring in cancers [12]. Reduced levels of global DNA methylation provoke genomic instability and thus promote carcinogenesis, while promoter hypermethylation usually results in transcriptional gene inactivation [13]. The DNA methylation status and individual susceptibility to cancers could be related to changes in the activities of folate metabolism enzymes derived by genetic polymorphisms.

In this regard, it has been shown that gene polymorphisms of the enzyme 5,10-methylenetetrahydrofolate reductase (*MTHFR*) affect the levels of available endogenous folates. MTHFR catalyzes the reduction of 5,10-methylenetetrahydrofolate to 5-methyltetrahydrofolate, the methyl group donor for homocysteine remethylation to methionine, which in turn is converted to *S*-adenosyl-l-methionine (SAM), the main donor of methyl groups in different methylation reactions [14]. MTHFR activity may be affected by two common single-nucleotide substitutions resulting in amino acid changes, namely C677T (Ala222Val) and A1298C (Glu429Ala), and can vary significantly between different individuals [15,16,17]. The *MTHFR* C677T polymorphism affects the enzyme’s thermal stability, resulting in the reduced activity of this enzyme [14]. TT677 individuals exhibit about a 50% reduction in enzyme activity and have increased plasma homocysteine concentrations as well as an altered balance of folate metabolites [16,18]. The A1298C polymorphism affects enzyme function to a lesser degree [18,19], but when accompanied by the C677T polymorphism has a more powerful effect on the decrease of MTHFR enzyme activity [20]. 

Although several studies investigated the influence of *MTHFR* polymorphisms on aberrant DNA methylation, to date inconsistent data are available [21]. Previous studies demonstrated that the *MTHFR* C677T polymorphism influences global DNA methylation status through an interaction with the folate status [22,23]. On the other hand, the study of de Arruda and collaborators [24] showed no significant effects of the *MTHFR* C677T polymorphism on the global DNA methylation of oral epithelial cells obtained from healthy subjects. 

*p16* and *MGMT* are important genes coding for proteins that play significant roles in carcinogenesis. *p16*, a cyclin-dependent kinase-4 inhibitor, is a tumor suppressor protein and the *MGMT* gene encodes *O*-6-methylguanine-DNA methyltransferase, an enzyme involved in DNA repair. One of the mechanisms leading to their inactivation is the aberrant hypermethylation of their promoter regions. *p16* is hypermethylated across many tumor types including colorectal, lung, and breast carcinomas [25]. 

The aim of this study was to assess the influence of the *MTHFR* C677T and A1298C polymorphisms on global DNA methylation and site-specific methylation of *p16* and *MGMT* gene promoters in a cohort of patients with OSCC compared with healthy subjects.

## 2. Results

Genotyping of patients and controls for *MTHFR* C677T and A1298C gene polymorphisms showed that the observed and expected genotype frequencies were in Hardy–Weinberg equilibrium in both groups (C677T: *p* = 0.16 for patients, *p* = 0.37 for controls; A1298C, *p* = 0.032 for patients, *p* = 0.06 for controls).

The frequency of the T677 mutated allele was significantly higher in patients than in controls (0.6 vs. 0.38, *p* = 0.00015), while the frequency of the *MTHFR* C1298 mutated allele was similar between cases and controls (0.23 vs. 0.28). In particular, the TT677 genotype was significantly more frequent in patients than in control subjects (31% vs. 16.6%, *p* = 0.04), while the CT677 genotype frequency only tended to be significantly higher in patients than in controls (58.6% vs. 42.2%, *p* = 0.064). The CC677 wild-type genotype was significantly less frequent in patients than in control subjects (10.4% vs. 41.1%, *p* < 0.0001).

No significant differences were found in the genotype distribution for the *MTHFR* A1298C polymorphism between the two groups. The wild-type AA1298 genotype was the most frequent both in patients and controls, accounting for more than half the population (55% vs. 63.3%, *p* = 0.39). The AC1298 heterozygous genotype was present in around one-third of the recruited patients and controls (34.6% vs. 27.7%, *p* = 0.46), while the CC1298 homozygous was mutated only in around 10% of the recruited subjects (10.3% vs. 7.7%, *p* = 0.78). 

The distribution of *MTHFR* genotypes in OSCC patients and healthy subjects is shown in Table 1. Interestingly, the CT/AC and TT/AA genotypes were found to have similar frequencies, and to be significantly more prevalent in cases than in controls. The CC/CC and CT/AA genotypes had a similar distribution in the two groups, while the CC/AA and CC/AC genotypes were not found among cases. 

We next examined the DNA global and site-specific methylation status in patients and control subjects. No significant differences between patients and controls were found with regard to the total content of methylated cytosines (3.61% vs. 3.43%, *p* > 0.05). Instead, the analysis of site-specific methylation revealed that about half of cases exhibited either a *p16* or *MGMT* promoter region methylated with a significantly higher frequency in comparison to the controls (44.8% vs. 13.4%, *p* < 0.0001). The promoter region of the MGMT gene was methylated in a higher number of patients in comparison with that of the *p16* gene (Table 2). 

The methylation of either the *p16* or *MGMT* promoter region was associated with a little over a three-/four-fold increase of risk for OSCC, as shown by the odds ratio (OR) calculation (Table 2). Notably, the methylation on the both *p16* and *MGMT* promoter regions was not observed among healthy subjects, while it was present in one-fifth of patients, and was associated with an increase of around 50-fold for OSCC; however, this latter result has to be considered with caution given the large interval size (Table 2).

We also evaluated whether the *MTHFR* genotype would affect the DNA methylation status in OSCC patients. Given the relatively small size of the six groups, including individuals with the same *MTHFR* genotype, we decided to put in one group, N (normal), 20 patients with the *MTHFR* CC/AA, CC/AC, CC/CC, and CT/AA genotypes, and in a second group, R (risk), 38 patients with the CT/AC and TT/AA genotype, which are known to be genetic determinants for alterations of MTHFR enzyme activity. 

After stratification of patients based on the *MTHFR* genotype, no significant differences were observed for global DNA methylation between the two groups. The mean content of methylated cytosine was about 3.6% in both groups.

Interestingly, the analysis of gene-specific methylation showed that the frequency of *p16* methylation was significantly higher in group R (22/38 subjects, 57.9%) than in group N where it was not observed (20/20 unmethylated subjects) (Table 3). Similarly, the frequency of *MGMT* methylation in group R (22/38 subjects, 57.9%) was higher in comparison to group N (6/20 subjects, 30%), even if this difference only tended to statistical significance (57.9% vs. 30%, *p* = 0.056) (Table 3).

## 3. Discussion

Several studies suggested that aberrant methylation of DNA has an important role in the development of several cancers, such as colorectal cancer, renal cancer and esophageal squamous cell cancer [26,27,28]. 

Researchers have proved that DNA methylation is related to age, diet, and other environmental factors [29,30,31]. In addition, the individual genetic background can affect the methylation status of DNA. Folate metabolism enzymes, such as MTHFR, methionine synthase and thymidylate synthase, are involved in the methylation process of DNA, and alterations in their activities could be a potential link between one-carbon metabolism and cancer development [32,33]. 

The association of the *MTHFR* C677T polymorphism with the risk for the development of several human cancers has been reported [34,35], although findings on its role in head/neck and oral cancer risk are inconsistent [36]. Indeed, the *MTHFR* C677T polymorphism, having major effects on MTHFR enzyme activity, was associated with an increase of oral cancer risk [37], while other studies showed a decreased risk for HNSCC and oral squamous cell carcinomas (OSCC) [38,39]. In our study, we observed that the presence of the T677 mutated allele was significantly higher in HNC patients, indicating that this polymorphism may play a role in oral cancer carcinogenesis. 

The role of the *MTHFR* A1298C polymorphism in cancer risk is less investigated, and in certain cancers it seems to play a protective function [40]. In our study the distribution of the C1298 mutated allele was similar between patients and control subjects, even if individuals bearing the C allele, namely those having the CC/AC and CT/AC genotypes, accounted for about 45%, while these same *MTHFR* genotypes were present only in 35% of controls. However, these differences were not significant. Interestingly, the frequency of the CT/AC genotype was higher than that of TT/AA (34.5% vs. 31%). The frequencies of these latter genotypes were significantly higher in patients than in controls. Noteworthy, among patients there were no subjects carrying the wild-type genotype for both polymorphisms (CC/AA). Although further studies should be carried out to better clarify the involvement of *MTHFR* polymorphisms, these data suggest the possible involvement of *MTHFR* C677T and A1298C in cancer development. 

To characterize the functional mechanism by which MTHFR polymorphisms may contribute to the development of OSCC in relation to the DNA methylation status, we also investigated the impact of these polymorphisms on both global and site-specific DNA methylation. 

Conflicting results on global DNA hypomethylation and the risk of cancer have been reported. Studies investigating DNA methylation alterations in HNC tissues showed the loss of global DNA methylation when compared to their matched normal adjacent tissues [41,42,43]. Instead, although some studies found a relationship between decreased global methylation in blood samples and cancer development [44], other studies stated that the methylation levels in tumor- and blood-derived DNA were independent [42]. This suggests that the evaluation of global DNA methylation in the cancer tissue may be more relevant for risk assessment. 

Interestingly, Subbalekha and collaborators [45] described similar hypomethylation levels in cells collected from oral rinses and OSCC tissues. In line with this observation, in our study we evaluated DNA methylation in saliva, a proxy tissue that may be useful as a biological matrix to identify subjects with a high risk of cancer development in a non-invasive manner. Saliva samples were collected prior to intervention to obtain methylation data referring to cancer tissues rather than the general individual methylation status. However, the global DNA methylation content was similar in saliva samples obtained from patients and control subjects, and was not affected by the MTHFR genotypes. 

Usually, LINE-1 hypomethylation measurements, which estimate a limited part of the genome, are employed to evaluate global DNA methylation. In our study, we used the capillary electrophoresis method which is able to detect the methylation of the whole genome, giving a more accurate evaluation than LINE-1 methylation. However, a limitation of this study is that the levels of cancer-specific methylation reported here may be underestimated due to the inability to distinguish the signal coming from the small percentage of tumor cells in saliva samples.

Of note, significant differences were observed when evaluating site-specific methylation on the *p16* and *MGMT* promoter regions. Promoter hypermethylation in the major genes involved in cell cycle, DNA damage repair, and cancer-related signaling pathways has been extensively studied in human cancers, including ESCC [46,47,48]. Among cell cycle–related genes, *p16* negatively regulates the G1-S transition in the cell cycle and has been found frequently methylated in precursor lesions of the esophagus, and thus *p16* function inactivation by hypermethylation is believed to be involved in the early stages of esophageal carcinogenesis [49]. Also the loss of function of DNA repair genes is associated with genomic instability and carcinogenesis. Although up to 130 genes are associated with DNA repair [50], *MGMT* is the major gene in the pathway of DNA repair and has been frequently found to be silenced by CpG island hypermethylation in many cancers, including esophageal adenocarcinoma [51].

Our study showed a higher frequency of *p16* and *MGMT* promoter methylation in patients diagnosed with OSCC than normal controls. About 50% of cases showed methylation of at least one gene and one-fifth had both *p16* and *MGMT* gene promoters methylated. In contrast, the concomitant methylation of *p16* and *MGMT* gene promoters was not observed in the controls. Overall, these results corroborate the concept that DNA methylation plays a major role in oral cancer development.

Recently, there has been growing interest to identify factors that can affect the patterns of DNA methylation. The MTHFR is an important enzyme in the one-carbon metabolism pathway that regulates the availability of methyl groups for methylation reactions, and several experimental and epidemiologic studies showed that the *MTHFR* C677T polymorphism may influence the DNA methylation status [21,23,52]. It has been shown that patients with the *MTHFR* TT677 genotype have a high risk of DNA hypermethylation in cancer tissues. However, only few studies carried out on a Chinese population evaluated the association of the *MTHFR* C677T polymorphism with aberrant CpG island hypermethylation of cancer-related genes, such as *p16* and *MGMT*, in patients with HNC. The authors showed that the aberrant hypermethylation of *p16*, *MGMT*, and *hMLH1* promoter genes was associated with the clinical characteristics of esophageal squamous cell cancer, and individuals carrying the *MTHFR* CT677 or TT677 genotype had a higher frequency of hypermethylation in the *MGMT* gene in cancer tissues [21,53].

On the other hand, another study failed to find a relationship between aberrant DNA methylation of genes, such as *p16*, *MGMT* and *hMLH1*, and the *MTHFR* C677T polymorphism in ESCC [28]. The discrepancy of these results might be due to the study design, the source of subjects and the sample size. Most importantly, these studies did not take into account the synergistic effects of the two *MTHFR* C677T and A1298C gene polymorphisms. Indeed, to our knowledge, this is the first study evaluating the effects of *MTHFR* genotypes at genetic loci C677T and A1298C on *p16* and *MGMT* gene promoter methylation in OSCC. 

Interestingly, we found that the *p16* gene promoter was methylated in around 60% of patients having either the CT/AC or TT/AA genotype, while it was unmethylated in patients having either the *MTHFR* CC/AA, or CC/AC, or CC/CC, or CT/AA genotype, and this difference was highly significant. The frequency of *MGMT* promoter methylation was also found to be higher, but not statistically significant, in patients with the CT/AC and TT/AA genotypes. The lack of significance for *MGMT* could be due to the small sample size analyzed. 

Overall, our findings suggest that the *MTHFR* polymorphisms may have an important role in OSCC carcinogenesis, probably due to their influence on gene-specific methylation processes.

The potential mechanism by which a reduction in MTHFR activity induced by C677T and A1298C polymorphisms may affect site-specific methylation has not been investigated yet. It has been previously hypothesized that some genes could have different dosage sensitivity to alterations in methyl-donor availability in comparison to others, and the dosage sensitivity could also be dependent on the tissue type and gene-environment interactions [54]. 

However, further studies on a larger population also evaluating other possible factors acting on the one-carbon metabolism, such as the folate levels, are needed for a more extensive understanding of the regulation of the methylation process by *MTHFR* polymorphisms. In addition, given the small sample size, we could not make any association with the tumor staging.

Even if similar previous studies have documented the association of *MTHFR* polymorphism and gene promoter hypermethylation, the novelty of the present study is the use of salivary samples to obtain data. In fact, based on the recently developed molecular detection methods, circulating tumor DNA can now easily be extracted from serum, plasma, saliva, broncho-alveolar lavage fluid, and urine [55]. Moreover, given that DNA methylation has been reported as an early event during carcinogenesis, and can be detected in different body fluids, the assessment of the DNA methylation status may represent a powerful diagnostic approach for cancer early detection [56]. Therefore, the use of simple and non-invasive tools to monitor the methylation status in patients would be highly desirable.

## 4. Materials and Methods

### 4.1. Study Subjects

Fifty-eight (40 M, 18 F; 50.2 ± 8.6 years) patients with oral squamous cell carcinoma (OSCC), who referred for diagnosis to the Division of Odontostomatology at Polyclinic Hospital University of Messina, were recruited for this study. Twenty-two of them were smokers. No alcohol use was reported among patients.

In the same time period, 90 healthy subjects (62 M, 28 F; 44.6 ± 11.3 years), matched with patients for age, gender, and smoking habit, were recruited on a voluntary basis among staff of Polyclinic Hospital University. Saliva samples were collected with the Oragene^®^ DNA Self-Collection kit (Genotek, Ottawa, ON, Canada) from all participants, before any interventions occurred. 

All subjects gave their informed consent for inclusion before they participated in the study. The study was conducted in accordance with the Declaration of Helsinki, and the protocol was approved by the Ethics Committee of the Polyclinic University of Messina (Project identification code: 12/16, 22 March 2016).

### 4.2. MTHFR Genotyping

Genomic DNA was purified from saliva using Oragene DNA kit (Genotek’s), according to manufacturer’s instructions. Genotyping for *MTHFR* C677T and A1298C polymorphisms was carried out by a Real-Time PCR allelic discrimination technique, using Pre-designed TaqMan SNP Genotyping Assays (Applied Biosystems; assay ID: C_1202883_20 and C_850486_20).

### 4.3. DNA Methylation Detection

For the analysis of global DNA methylation the extracted DNA was hydrolyzed by 90% formic acid. After hydrolysis, samples were evaporated and the dry residue containing free bases was dissolved in ultrapure water and immediately analyzed by capillary electrophoresis as described previously [57]. The percentage of methylated to total cytosine (mC/tC) was calculated using the formula: (mmol mC/(mmol mC + mmol C)) × 100. As previously demonstrated, capillary electrophoresis with short-end injection mode resulted in the method sensitivity enhancement. Therefore, these procedures were comparable to others assays [57]. All assays were performed in duplicate.

The methylation at the promoter region of *p16* and *MGMT* genes was determined by methylation-specific PCR (MSP) after sodium bisulfite modification of DNA using a commercial kit from Sigma, according to the manufacturer’s instructions. Each MSP reaction was carried out in triplicate. The sequences of primer pairs used in MSP are shown in Table 4. PCR products were loaded onto 3.0% gels, stained with ethidium bromide, and directly visualized under UV illumination.

### 4.4. Statistical Analyses

Statistical analysis was performed with SPSS statistical program version 13.0 (SPSS, Chicago, IL, USA). Differences in the distribution of *MTHFR* genotypes as well as methylation status among patients and controls were examined by using the χ^2^ test or Fisher’s exact test, where appropriate. A value of *p* < 0.05 was considered statistically significant.

## Figures and Tables

**Table 1:** Distribution of *MTHFR* genotypes in OSCC patients and healthy subjects.

Genotype	Cases (*n* = 58) (%)	Controls (*n* = 90) (%)	*p*
CC/AA	-	21 (23.3)	<0.0001
CC/AC	-	8 (8.8)	0.019
CC/CC	6 (10.3)	8 (8.8)	0.76
CT/AA	14 (24)	21 (23.3)	0.9
CT/AC	20 (34.5)	17 (18.8)	0.032
TT/AA	18 (31)	15 (16.6)	0.04

**Table 2:** Analysis of site-specific methylation on *p16* and *MGMT* promoter regions in OSCC patients and controls.

Gene	Cases (*n* = 58) (%)	Controls (*n* = 90) (%)	*p*	Odds Ratio (95% CI)
*p16*	10 (17.2)	5 (5.6)	0.027	3.54 (1.143–10.97)
*MGMT*	16 (27.6)	7 (7.8)	0.002	4.52 (1.72–11.83)
*p16* + *MGMT*	12 (20.7)	-	<0.0001	48.66 (2.82–840.7)

**Table 3:** Analysis of the influence of the *MTHFR* genotype on either *p16* gene promoter methylation or *MGMT* promoter methylation in OSCC patients.

	*MTHFR* Genotype		
	Normal	Risk	*p*
*p16* methylated	0 (0%)	22 (57.9%)	<0.0001
*MGMT* methylated	6 (30%)	22 (57.9%)	0.056

**Table 4:** Sequences of primers used in methylation-specific PCR.

Primer	Forward 5′ > 3′	Reverse 5′ > 3′	Tm (°C)
p16-UM	TTATTAGAGGGTGGGGTGGATTGT	CAACCCCAAACCACAACCATAA	58
p16-M	TTATTAGAGGGTGGGGCGGATCGC	GACCCCGAACCGCGACCGTAA	55
MGMT-UM	TTTGTGTTTTGATGTTTGTAGGTTTTTGT	AACTCCACACTCTTCCAAAAACAAAACA	60
MGMT-M	TTTCGACGTTCGTAGGTTTTCGC	GCACTCTTCCGAAAACGAAACG	60
